# Diet‐induced obesity results in impaired oral tolerance induction

**DOI:** 10.1002/iid3.720

**Published:** 2022-11-18

**Authors:** Katharina Streich, Margarethe Klein, Anja Siebert, André Bleich, Manuela Buettner

**Affiliations:** ^1^ Institute for Laboratory Animal Science Hannover Medical School Hannover Germany; ^2^ Institute for Neurophysiology Hannover Medical School Hannover Germany

**Keywords:** lymph nodes, stromal cells, transplantation

## Abstract

**Introduction:**

Obesity increases the risk of several diseases, such as type 2 diabetes mellitus and cardiovascular disease. Obesity also affects the immune system. When dietary lipids are transported via the lymphatics, they pass the mesenteric lymph nodes (mLNs). In these secondary lymphoid organs, immune responses towards pathogens are generated, or tolerance against harmless antigens is induced.

**Methods:**

In this study, the effects of diet‐induced obesity (DIO) on mLN induced oral tolerance induction were examined in C57BL/6NCrl mice. Therefore, mice were fed a high‐fat or a low‐fat diet for 14 weeks. After 10 weeks of feeding oral tolerance induction started, ending up in measuring the delayed‐type hypersensitivity reaction, the cell subset composition and cytokine expression.

**Results:**

We detected an impaired oral tolerance induction during DIO, but changes were reversible after switching the feed to standard chow. Thus, the altered immunological function of mLNs depends on the intake of dietary lipids. Additionally, our results show an influence of the microenvironment on the development of oral tolerance during DIO as oral tolerance was induced in transplanted peripheral lymph nodes.

**Conclusion:**

This indicates a functional influence of dietary lipids on stromal cells involved in immune system induction in the mLNs.

## Graphical abstract

Our findings indicate an altered oral tolerance induction during obesity. Scientists interesting in obesity and/or oral tolerance will be interested in a disturbed lymph node function after high fat diet

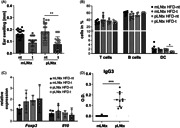

## 1 INTRODUCTION

Obesity is a disease with an increasing prevalence worldwide.^1^, ^2^, ^3^ One of the major drivers of obesity is unhealthy dietary habits and reduced physical activity.^2^, ^3^ Lipids and other nutrients are absorbed by enterocytes in the intestine^4^, ^5^, ^6^ and are transported as chylomicrons via the lymphatics into the blood.^4^, ^5^ On their way, they pass through the mesenteric lymph nodes (mLNs), which drain the intestinal tract.^7^, ^8^

mLNs are secondary lymphoid organs in which immune cells are activated during infection or regulated for the induction of tolerance.^9^, ^10^ As lipids are transported via lymph fluid through the mLNs during obesity, they come into contact with various cells.^7^ We recently showed that almost exclusively, stromal cells and macrophages are involved in lipid uptake in the mLNs.^7^ Because all these cells are involved in immune response induction or homeostasis maintenance, we assume that high lipid intake, lipid transport through the sinuses of the LNs and lipid absorption impair the immunological function of these cells.

Oral tolerance takes place in the area of the intestine, and it is an important peripheral tolerance process.^11^ The intestinal mucosa is confronted with a multitude of bacteria, food components and pathogens every day. The intestinal immune system must therefore be able to differentiate very precisely between dangerous and harmless antigens.^12^ To this end, the intestinal immune system induces an active immune response against harmful pathogens, while it has the ability to develop tolerance to harmless foreign antigens, such as commensal bacteria or food antigens. However, it was shown that the antigen dose determines which peripheral tolerance mechanisms are effective. For example, a single high dose of protein antigens leads to anergy and/or deletion of antigen‐specific T cells. In contrast, repeated application of small amounts of protein antigens is responsible for the formation of regulatory T cells (Tregs).^13^ It was later shown that an increased antigen dose leads to improved induction of Tregs.^14^ The mechanism of deletion and/or anergy could therefore also contribute to tolerance induction. This mainly takes place in the mLNs.^10^ Furthermore, the microenvironment of the LN is also involved in tolerance induction: stromal cells as well as dendritic cells in the mLNs can express retinoic acid‐producing enzymes (RALDH2) and both together lead to the differentiation to Foxp3 Tregs. In contrast, the microenvironment of peripheral lymph nodes (pLN) transplanted into the mesentery induce oral tolerance via IgG3 producing cells.^15^

The effects of oral tolerance are typically determined systemically based on a hyporesponsiveness of the delayed type (delayed‐type hypersensitivity [DTH] reaction), reduced T‐cell proliferation and reduced proinflammatory cytokine production. This is mainly due to CD4+ T cells, as depletion can reverse these effects.^16^ Adoptive transfer of these cells into a naive individual can also transmit tolerance.^17^ However, the impact of diet‐induced obesity (DIO) on oral tolerance or immune response induction in the mLNs is still largely unknown.

## 2 MATERIALS AND METHODS

### 2.1 Mice

Male C57BL/6NCrl mice at the age of 7–8 weeks were bred at the central animal laboratory of Hannover Medical School. All experimental animals were kept under standardized conditions at the Central Animal Laboratory of the MHH. The rooms had controlled environmental conditions (room temperature: 21 ± 2°C; relative humidity: 60 ± 10%; lighting cycle: 14 h light: 10 h dark; air changes: 12–14 per hour). The animals were kept in a conventional group housing of two to five animals in type II L cages (Uno) on softwood pellets, which were renewed once a week. The animals were provided with water and pelleted feed ad libitum.

The experimental animals used were regularly and routinely monitored for health in accordance with the recommendations of the Federation of European Laboratory Animal Science Associations.^18^ The mice tested positive for *Pasteurella pneumotropica, Helicobacter spp., β‐haemolytic streptococci, Klebsiella oxytoca, Staphylococcus aureus*, murine norovirus, *Trichomonas spp.* and *apathogenic intestinal flagellates* as opportunistic pathogens.

### 2.2 Monitoring and grouping

Mice were randomized into 4 groups for feeding experiments (Table 1) and 8 groups for transplantation experiments. Each group consisted of 4–14 animals.

**Table 1:** Experimental groups

	Tolerance induction	
Diet	**PBS (not tolerized)**	**OVA (tolerized)**
LFD	LFD‐nt	LFD‐t
HFD	HFD‐nt	HFD‐t

Abbreviations: HFD, high‐fat diet; LFD, low‐fat diet; PBS, phosphate‐buffered saline.

The health status as well as the weight of the mice were checked at least twice a week. After surgery, the weight of the animals was checked daily for a period of 1 week and the clinical condition was classified using a score. A clinical score below 2 (no activity, transition to moribund) was defined as a criterion for termination, which resulted in immediate euthanasia.

### 2.3 Feeding

Mice were fed a high‐fat diet (HFD; #D12492, Research Diets) containing 60% kcal fat or a matched low‐fat diet (LFD; #D12450J, Research Diets) that contained 10% kcal fat ad libitum for 14 weeks. Analyzing the influence of the diet on the DTH response, mice were fed a HFD or LFD for 10 weeks, and afterwards, they received standard chow (SD; #132003) until the end of the experiment.

### 2.4 Intestinal surgery

As described earlier,^19^ mLNs and pLNs were isolated from male C57BL/6NCrl mice and used as donors for male C57BL/6NCrl mice. Under combined anesthesia with ketamine (Anesketin® 100 mg/ml; 100 mg/kg) and xylazine (Rompun® 20 mg/kg; 2.8 mg/kg KGW), the mLNs of the small and large intestine of the host were removed, and donor mLNtx or pLNtx (axillary, brachial, inguinal and popliteal) LNs were transplanted into this region.

### 2.5 Oral tolerance induction

After transplantation and/or 10 weeks of feeding, mice were fed by gavage with 25 mg of OVA (Grade III; Sigma‐Aldrich) in 200 µl of phosphate‐buffered saline (PBS) or PBS only as a control on days 0, 3, 6, and 8 for tolerance induction. On day 16, mice were immunized by subcutaneous injection of 300 µg of OVA (Grade VI; Sigma‐Aldrich) in 200 µl of PBS emulsified in complete Freud's adjuvant (Sigma‐Aldrich). On day 34, mice were challenged by subcutaneous injection of 50 µg of OVA (Grade VI) in 10 µl of PBS into the right ear and PBS only into the left ear. Ear swelling was measured before challenge and 48 h later. The DTH response was calculated as followed: (right ear thickness − left ear thickness)^48 h^ − (right ear thickness − left ear thickness)^0 h^.

### 2.6 Quantitative real‐time PCR (RT‐qPCR)

Total RNA from the mLNs was isolated using the RNeasy® Mini Kit (Qiagen). For this purpose, the mLNs were taken up in RLT buffer with 2% dithiothreitol (DTT) and homogenized using an ultrasound processor. Further purification was carried out according to the manufacturer's instructions with an additional step of DNase digestion (RNase‐Free DNase Set; Qiagen). The present RNA concentration was then measured using a spectrophotometer (NanoDrop® spectrophotometer), and the samples were stored at −80°C. Complementary DNA (cDNA) was synthesized by using a QuantiTect Reverse Transcription Kit (Qiagen) according to the manufacturer's protocol. The cDNA obtained for RT‐qPCR was performed using TaqMan® Fast Advanced Master Mix and TaqMan® Gene Expression Assays for *Il10* (Mm01288386_m1) and *Foxp3* (Mm00475162_m1), as well as *β‐actin* (Mm00607939_s1) as an endogenous control (all acquired from Thermo Fisher Scientific). Gene expression was determined in a StepOnePlus™ Real‐Time PCR System (Applied Biosystems). All reactions were run in triplicate. Relative gene expression was calculated in relation to a reference sample using the 2^−ΔΔCt^ method.

### 2.7 Flow cytometry

The extracted mLNs were treated with 3 ml of a collagenase solution (collagenase from Clostridium histolyticum, type VIII; 0.75 mg/ml) and incubated for 10 min at 37°C. After this incubation and centrifugation, the supernatant was discarded, while the cell pellets were resuspended and separated through a cell sieve (70 μm). Afterwards the immune cell subset composition was analyzed by flow cytometry. The Following antibodies were used: CD3‐APC‐Cy7 (clone 145‐2C11), CD103‐PE (clone 2E7), MHCII‐PE‐Cy7 (clone M5/114.15.2) (all acquired from Biolegend); B220‐VioBlue® (clone RA3‐6B2, acquired from Miltenyi); and CD11c‐APC (clone HL3, BD Biosciences). Cytokine and FOXP3 staining was performed after surface staining with CD90‐APC‐Cy7 (clone 30‐H12), CD4‐FITC (clone GK15), CD25‐APC (clone PC61) (all acquired from Biolegend). For intracellular stainings, cells were washed twice and treated with a True‐Nuclear™ Transcription Factor Buffer set (BioLegend) according to the manufacturer's instructions, including incubation with FOXP3‐Pacific blue (clone MF‐14, BioLegend). Flow cytometric analysis was performed using a flow cytometer (Gallios™, Beckmann Coulter) and Kaluza Analysis 1.3 software (Beckmann Coulter).

### 2.8 OVA‐specific **enzyme‐linked immunoassay** (ELISA)

After measuring the DTH response mice were sedated by CO_2_ inhalation and subsequently killed by exsanguination through cardiac puncture. Collected blood was centrifuged for 6 min at 500*g* to obtain serum. The serum samples were diluted 1:50 with PBS and temporarily stored at 4°C. The 96‐well ELISA microplates (MICROLONTM, high‐binding; Greiner Bio‐One) were coated with 100 μl of ovalbumin solution (grade III; 0.5 mg/ml) per well and stored overnight at 4°C. The following protocol was carried out at room temperature by means of a washing process with TBS‐T buffer between the individual steps. First, all unbound areas were blocked using 200 μl of a milk powder TBS‐T solution (5%) per well for 1 h at 37°C. After removing the supernatant, 100 μl of diluted sample was added to the wells and incubated for 2 h at 37°C. All samples, including the negative control (PBS), were measured in doublets. This was followed by incubation with 100 μl of the respective detection antibody (anti‐IgG_3_), as well as the subsequent incubation with 100 μl of the horseradish peroxidase (HRP) enzyme for 1 h at room temperature. The antibody and HRP enzyme were diluted 1:250 with PBS. The wells were then mixed with 100 μl of TMB substrates A and B and incubated for 2 min. Sulfuric acid then served as a stop solution. The color reaction was measured on a VICTORTM X3 ELISA reader at a wavelength of 450 nm for 0.1 s. The measured optical density in the serum of the OVA‐tolerated mice was then subtracted from the mean optical density of the control animals.

### 2.9 Statistical analysis

Before starting the experiment the group size was calculated using a Power analysis. All statistical analyses were performed using GraphPad Prism®6 software. Data were tested for normality with the D'Agostino‐Pearson (*n* ≥ 8) normality test. For smaller sample sizes, the Shapiro–Wilk normality test (*n* ≥ 7) or Kolmogorov‐Smirnov test (*n* ≥ 5) was used. Quantitative two‐group parametric data were analyzed with a *t* test, whereas data from at least three groups were analyzed by one‐way analysis of variance (ANOVA) with Tukey's test for multiple comparisons. Nonparametric data for more than two groups were analyzed by the Kruskal–Wallis test with Dunn's multiple comparisons test. Comparisons of data with two factors were analyzed by using two‐way ANOVA with Sidak's multiple comparisons test. Significance levels were set at 5%.

## 3 RESULTS

### 3.1 Decreased tolerance induction in HFD mice

**Figure 1:**
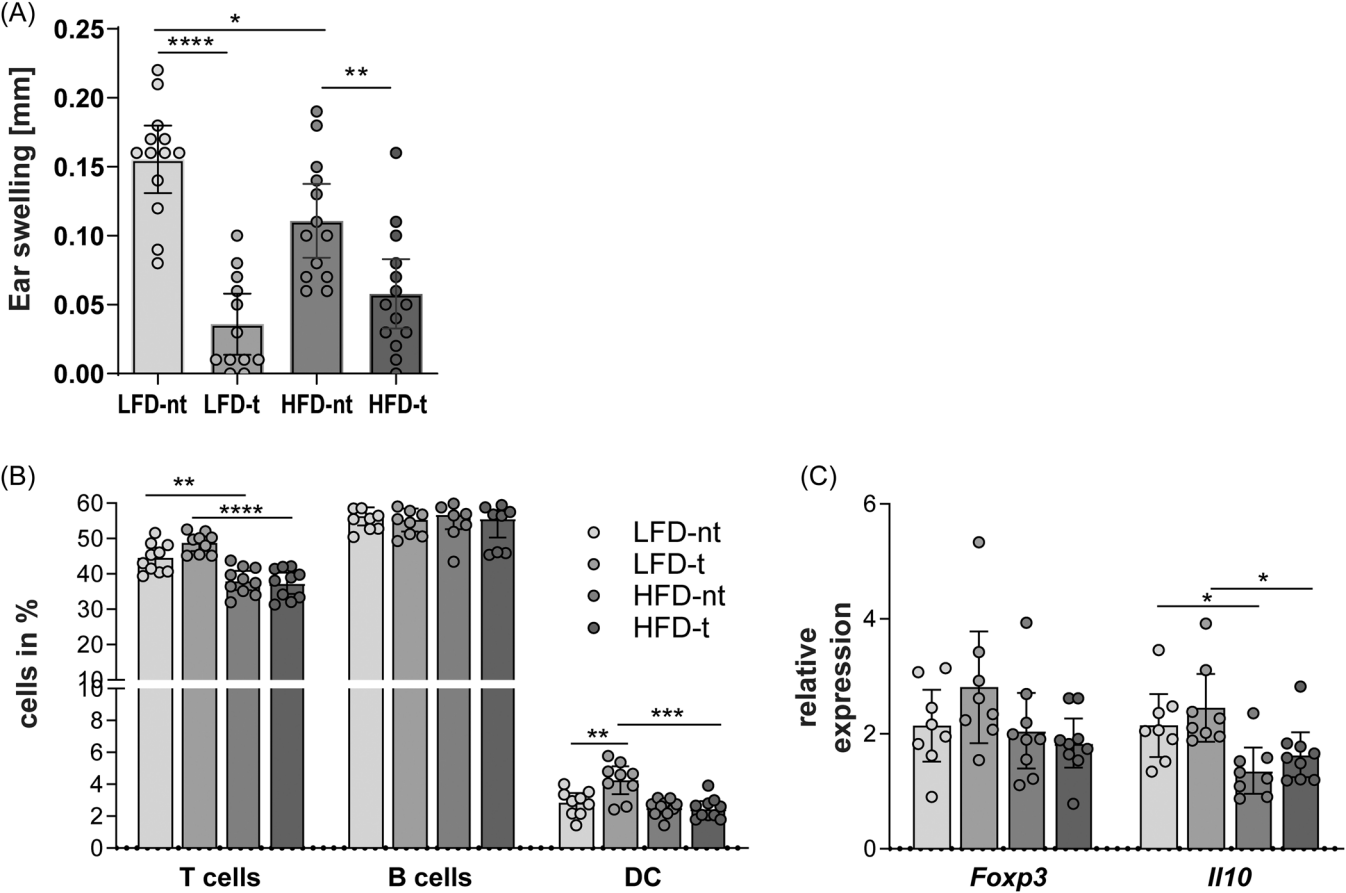
Hypersensitivity reaction in the combined DIO and tolerance test model. After induction of oral tolerance by feeding OVA to LFD‐ or HFD‐fed mice, (A) the DTH response, (B) immune cell populations, and (C) cytokine expression of the mLNs were analyzed (*n* = 8–14; mean ± 95% CI; one‐way‐ANOVA with subsequent Tukey's test or Mann–Whitney *U* test; ∗*p* < .05, ∗∗*p* < .01, ∗∗∗*p* < .001, and ∗∗∗∗*p* < .0001). Two independent experiments were performed. ANOVA, analysis of variance; CI, confidence interval; DIO, diet‐induced obesity; HFD, high‐fat diet; LFD, low‐fat diet.

One important function of the mLNs is the induction of oral tolerance. Therefore, mice were fed the LFD or HFD for 14 weeks. After 10 weeks of feeding, oral tolerance induction was started and finally measured by the DTH response (Table 1). The DTH reaction has been well characterized as an influx of immune cells and subsequent swelling at the Ag injection site.^20^, ^21^ As expected, tolerized mice showed reduced ear swelling and consequently induction of oral tolerance (Figure 1A). However, HFD‐nt mice showed significantly reduced ear swelling compared to that of LFD‐nt mice, whereas HFD‐t mice had a slightly enhanced DTH response compared to that of LFD‐t mice. These results reveal that DIO led to a reduced difference between tolerized and nontolerized mice during tolerance induction (Figure 1A).

Next, we were interested in differences in the immune cell population. We detected a reduced percentage of CD3+ T cells in the mLNs of HFD mice compared to LFD mice (Figure 1B), regardless of their treatment. The frequency of B cells was similar in the different groups (Figure 1B). In addition to lymphocytes, antigen‐presenting DCs play the most important role in successful tolerance induction. Tolerogenic DCs can be characterized by their expression of CD103.^22^, ^23^ A significantly higher proportion of these MHCII+ CD11c+ CD103+ cells was detected in the tolerized LFD mice than in the HFD mice (Figure 1B).

Furthermore, expression of the transcription factor *Foxp3* as well as the cytokine *Il10* in the mLNs was investigated by flow cytometry (Table 2) as well as qPCR (Figure 1C). No differences were detectable after intracellular staining of CD4+ T cells for their expression of FOXP3. In addition, analysis of the gene expression showed similar *Foxp3* levels in all groups, but lower levels of *Il10* were detected in HFD mice than in LFD mice (Figure 1C). Thus, reduced T cells and tolerogenic DCs as well as decreased *Il10* expression seem to be the cause for the impaired oral tolerance induction in HFD mice.

**Table 2:** FOXP3 expression on CD4 T cells using flow cytometry

	Tolerance induction			
Staining	LFD‐nt	LFD‐t	HFD‐nt	HFD‐t
	Mean; 95% CI	Mean; 95% CI	Mean; 95% CI	Mean; 95% CI
FOXP3	12.93; 9.55–16.31	13.50; 11.95–15.05	13.28; 10.00–16.57	11.63; 8.67–14.58

Abbreviations: CI, confidence interval; HFD, high‐fat diet; LFD, low‐fat diet.

### 3.2 Diet influences oral tolerance induction

**Figure 2:**
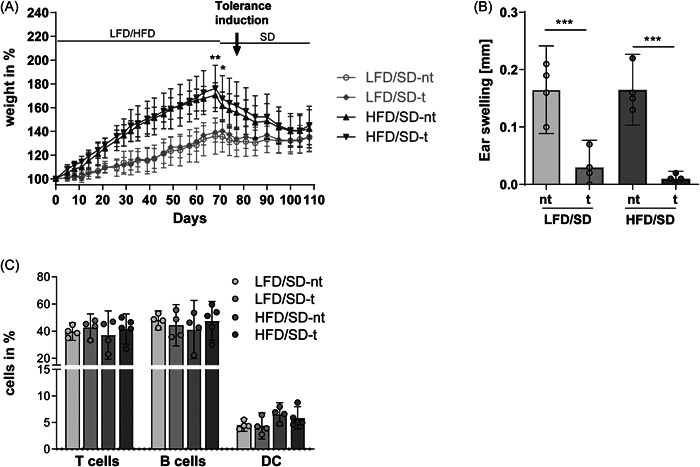
After 10 weeks of feeding a LFD or HFD, mice were fed standard chow (SD) until the end of the experiment. (A) Body weight was measured twice per week and calculated at the initiation of LFD or HFD feeding (in %; *n* = 25; mean ± 95% CI). (B) DTH response and (C) immune cell populations were analyzed (*n* = 4–5; mean ± 95% CI; one‐way‐ANOVA; ∗*p* < .05, ∗∗*p* < .01, and ∗∗∗*p* < .001). ANOVA, analysis of variance; CI, confidence interval; HFD, high‐fat diet; LFD, low‐fat diet.

The significant differences in the hypersensitivity response and the different cell populations within the mLNs of obese mice compared to control animals suggested a disturbance of tolerance induction. However, how strong the influence of diet on this pathological effect was and whether this effect was reversible remained unclear. Therefore, mice were fed a LFD or HFD for 10 weeks. Afterwards, mice were fed the SD until the end of the study. Tolerance induction was started 7 days after the change of the food. First, HFD mice showed an increasing body weight, but the change to the SD resulted in weight loss (Figure 2A). The systemic effect of tolerance induction was investigated using the hypersensitivity reaction (Figure 2B). The SD groups showed a significant difference between nontolerized and tolerized mice, independent of the previously fed diet. Changing the food to the SD resulted in a recovery of normal tolerance induction in the HFD mice. This indicates that the changes observed are reversible (Figure 2B).

Again, flow cytometric analyses using the same gating strategies and evaluations were carried out to clarify whether the change in diet can influence the cell subset composition of the mLNs. Interestingly, similar frequencies of T cells, B cell and tolerogenic DCs were detected in all groups, independent of diet or tolerance (Figure 2C). Our results, particularly those obtained by the hypersensitivity reaction, indicate that a HFD diet can significantly influence oral tolerance induction. The previously reduced immune response could largely be restored by an intermediate change to the SD, suggesting a strong influence of diet on the immunological function of the mLNs.

### 3.3 Oral tolerance in pLN‐transplanted animals

**Figure 3:**
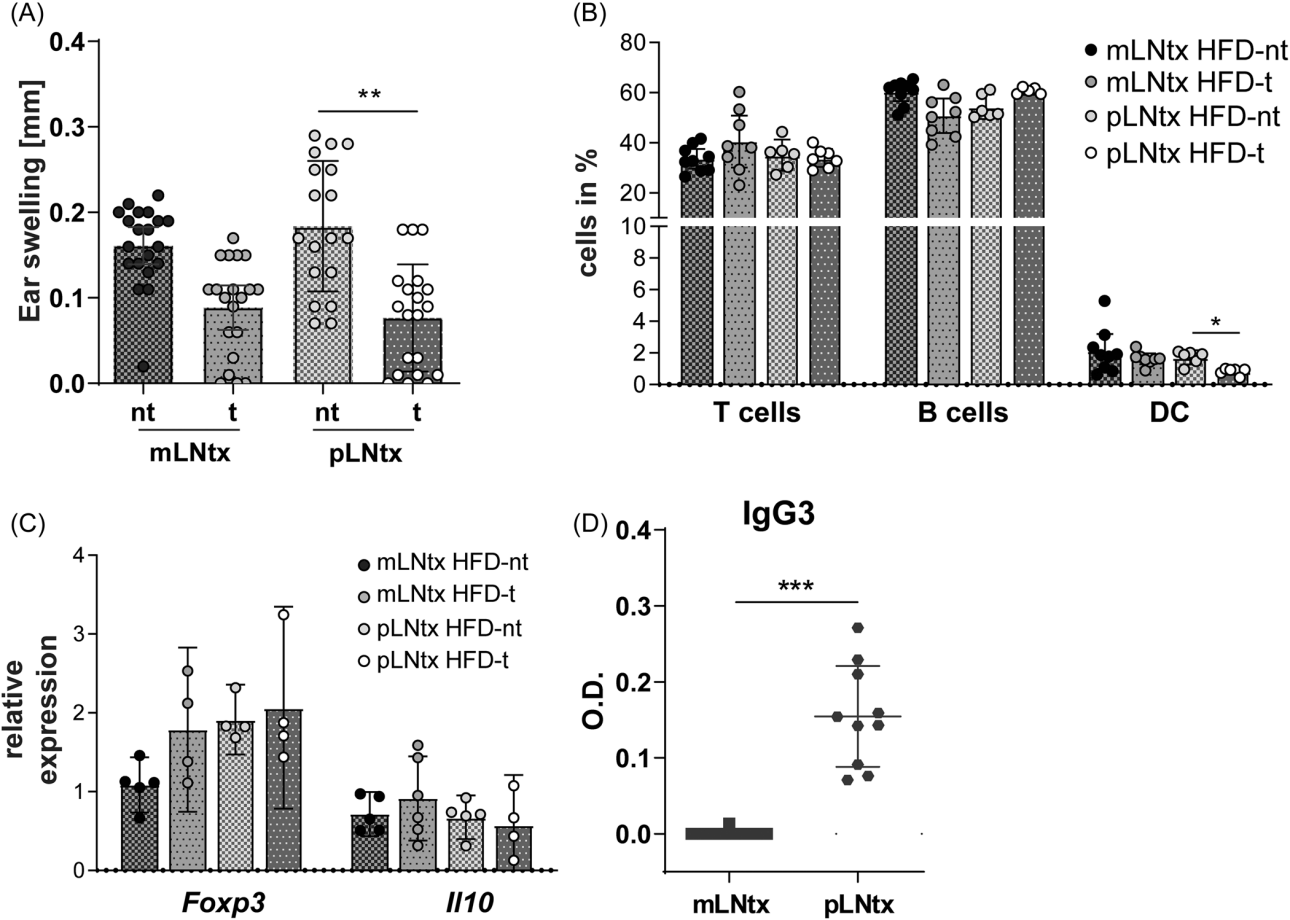
Transplantation of mLNs or pLNs was performed, and the mice were allowed to recover for 8 weeks. Afterwards, mice were provided with the HFD for 10 weeks, and induction of oral tolerance by feeding OVA started. Two independent experiments were performed. (A) The DTH response, (B) immune cell populations of LNtx, and (C) Gene expression analysis of the pLNtx and mLNtx mice after providing the HFD and inducing oral tolerance. Relative gene expression of cytokines in LNtx was measured by qPCR and normalized to a reference sample set to 1 (*n* = 3–6, each dot represents the mean of 3 technical replicates; mean ± 95% Cl). (D) Serum via an Ag‐specific Ig ELISA were analyzed (*n* = 8‐14; mean ± 95% CI; one‐way‐ANOVA with subsequent Tukey test or Mann–Whitney *U* test; ∗*p* < .05, ∗∗*p* < .01, and ∗∗∗*p* < .001). ANOVA, analysis of variance; CI, confidence interval; DTH, delayed‐type hypersensitivity; ELISA, enzyme‐linked immunoassay; HFD, high‐fat diet; LFD, low‐fat diet; mLN, mesenteric lymph nodes.

As previously shown, pLNtx‐transplanted animals were able to develop oral tolerance.^15^ However, the microenvironment of the LN can influence tolerance induction, as B cells are suggested to be important for tolerance induction in pLNtx mice.^15^ Here, we wanted to analyze whether this method of induction is influenced by feeding a HFD. After LN transplantation and regeneration, mice were fed a HFD or LFD for 14 weeks. After 10 weeks of feeding, oral tolerance induction was started, and in the end, the DTH response was measured. As expected, the DTH response was reduced in the mLNtx‐LFD‐t mice compared to the nt control group but only a slight reduction was measured between pLNtx‐LFD mice (Figure S1A). When comparing the mLNtx‐HFD mice directly, no significant difference between the nontolerized and tolerized animals could be demonstrated (Figure 3A). This is probably due to the reduced DTH response of the nontolerized mLNtx‐HFD animals, similar to the reduced DTH response that was observed in nontolerized HFD mice (Figure 1A). Furthermore, a significant difference between the nontolerized and tolerized groups was identified in the pLNtx‐HFD mice. The results suggest a disturbance of mLN stromal cells due to increased lipid intake.

Flow cytometric analyses showed no differences in the T and B cell subsets between the nontolerized and tolerized mice after HFD feeding, independent of the transplanted LNs (Figure 3B and Figure S1B). Furthermore, no differences were detectable in FOXP3 expression in CD4+ T cells (Table 3). Finally, the immune cells were also analyzed by flow cytometry (Figure 3B), and decreased percentages within the population of tolerogenic DCs were detected in tolerized pLNtx mice, whereas no differences between the mLNtx groups could be identified. The expression levels of genes involved in regulatory responses, such as *Foxp3* and *Il10*, were similar in mLNtx and pLNtx mice (Figure 3C). To clarify to what extent detectable immunoglobulin‐positive cells produce OVA‐specific antibodies, serum was additionally examined using an OVA‐specific ELISA. In contrast to the HFD mLNtx‐t mice, increased concentrations of OVA‐specific IgG_3_ could be determined in the HFD pLNtx‐t mice (Figure 3D). In summary, these results show that the microenvironment has an impact on the induction of immune responses during obesity. The analysis showed systemic differences between the mLNtx and pLNtx mice during HFD feeding. This indicates a restricted function of the stromal cells within the mLN due to increased lipid intake.

**Table 3:** FOXP3 expression on CD4 T cells using flow cytometry

	Tolerance induction			
Staining	mLNtx HFD‐nt	mLNtx HFD‐t	pLNtx HFD‐nt	pLNtx HFD‐t
	Mean; 95% Cl	Mean; 95% Cl	Mean; 95% Cl	Mean; 95% Cl
FOXP3	29.89; 17.48–42.30	15.60; 6.72–24.48	17.98; −0.57–36.53	17.50; 4.10–30.90

Abbreviations: CI, confidence interval; HFD, high‐fat diet; LFD, low‐fat diet.

## 4 DISCUSSION

During obesity, increased dietary lipids are taken up and transported via lymphatic vessels and pass the mLNs. As we showed very recently, lymph node stromal cells and macrophages are affected, as these cells include increased numbers of lipid droplets.^7^ The aim of this study was to investigate the influence of DIO on the immunological function of mLN. MLNs are the only sites that can react to harmless food antigens or intestinal microflora with induction of tolerance. This oral tolerance is one of the most important peripheral tolerance mechanisms and was the focus of this work.

Therefore, we induced oral tolerance and measured the DTH response to ovalbumin in HFD and LFD animals. HFD‐nt mice showed a reduced hypersensitivity response compared to that of the control mice, while the HFD‐t mice showed a slightly increased response to ovalbumin administration. This indicates a disorder of the immune system and consequently a limited ability to induce tolerance. In another tolerance test model, no differences in the DTH response to ovalbumin could be found between C57BL/6 DIO mice and control mice.^24^ In this model, tolerance was achieved through a single subcutaneous application of ovalbumin.

In our study, we observed reduced T cells and tolerogenic DCs as well as decreased *IL10* expression, which suggests a reduced tolerogenic function of the mLNs after HFD feeding. Whether this reduced tolerogenic response is due to activation of fewer cells or to morphological LN changes has to be elucidated in further studies. However, this reduced immune response could largely be restored by an intermediate change in diet to the SD.

We previously showed that LNs implanted from the periphery into the mesentery consist of surviving stromal cells re‐establishing a functional LN with an LN‐specific microenvironment.^19^, ^25^ As transplanted pLNs were shown to induce oral tolerance via a B cell response,^15^ we also analyzed the capacity for tolerance induction of pLNs after HFD feeding. As expected LFD animals from mLNtx‐transplanted mice showed a decreased ear swelling after tolerance induction, but LFD fed pLN animals showed a higher variance. However, in HFD mice, oral tolerance was only induced in pLN‐transplanted mice, whereas mLN‐transplanted control mice showed impaired induction. In addition to previous findings, increased levels of antigen‐specific IgG3 were detected in pLNtx‐tolerized animals.

In summary, it was shown that DIO affects mLN induced oral tolerance induction. However, these changes are reversible after the change to standard chow. In addition, the microenvironment has an impact on the development of immune system induction during DIO. This indicates a functional influence of dietary lipids on stromal cells involved in tolerance induction in the mLNs.

## AUTHOR CONTRIBUTIONS

**Manuela Buettner**: conceived and designed the experiments. **Manuela Buettner, Margarethe Klein**: wrote the manuscript. **Manuela Buettner, Katharina Streich, Anja Siebert**: performed the experiments and analyzed the data. **Manuela Buettner and André Bleich**: supervised the work.

## CONFLICTS OF INTEREST

The authors declare no conflicts of interests.

## ETHICS STATEMENT

This study was conducted in accordance with German animal protection law and with the European Directive 2010/63/EU. All experiments were approved by the Local Institutional Animal Care and Research Advisory committee and permitted by the Lower Saxony State Office for Consumer Protection and Food Safety (LAVES; File Number: 13/1174).

## Supporting information

Figurementary Figure 1: Transplantation of mLNs or pLNs was performed, and the mice were allowed to recover for 8 weeks. Afterwards, mice were provided with the LFD or HFD for 10 weeks, and induction of oral tolerance by feeding OVA started. Two independent experiments were performed. A: The DTH response, B: immune cell populations were analyzed (n = 8‐14; mean ± 95% Cl; One‐way‐ANOVA with subsequent Tukey test or Mann‐Whitney‐U‐test).

## References

[1] Goossens GH . The metabolic phenotype in obesity: fat mass, body fat distribution, and adipose tissue function. Obes Facts. 2017;10(3):207‐215. 28564650 10.1159/000471488 PMC5644968

[2] Collaboration NCDRF . Rising rural body‐mass index is the main driver of the global obesity epidemic in adults. Nature. 2019;569(7755):260‐264. 31068725 10.1038/s41586-019-1171-x PMC6784868

[3] Malik VS , Willet WC , Hu FB . Nearly a decade on—trends, risk factors and policy implications in global obesity. Nat Rev Endocrinol. 2020;16(11):615‐616. 32873971 10.1038/s41574-020-00411-y PMC7461756

[4] Beilstein F , Carriere V , Leturque A , Demignot S . Characteristics and functions of lipid droplets and associated proteins in enterocytes. Exp Cell Res. 2016;340(2):172‐179. 26431584 10.1016/j.yexcr.2015.09.018

[5] Klop B , Elte JW , Cabezas MC . Dyslipidemia in obesity: mechanisms and potential targets. Nutrients. 2013;5(4):1218‐1240. 23584084 10.3390/nu5041218 PMC3705344

[6] Wang TY , Liu M , Portincasa P , Wang DQ . New insights into the molecular mechanism of intestinal fatty acid absorption. Eur J Clin Invest. 2013;43(11):1203‐1223. 24102389 10.1111/eci.12161 PMC3996833

[7] Streich K , Smoczek M , Hegermann J , et al. Dietary lipids accumulate in macrophages and stromal cells and change the microarchitecture of mesenteric lymph nodes. J Adv Res. 2020;24:291‐300. 32405435 10.1016/j.jare.2020.04.020 PMC7210474

[8] Kelch ID , Bogle G , Sands GB , Phillips ARJ , LeGrice IJ , Dunbar PR . High‐resolution 3D imaging and topological mapping of the lymph node conduit system. PLoS Biol. 2019;17(12):e3000486. 31856185 10.1371/journal.pbio.3000486 PMC6922347

[9] Hahn A , Thiessen N , Pabst R , Buettner M , Bode U . Mesenteric lymph nodes are not required for an intestinal immunoglobulin A response to oral cholera toxin. Immunology. 2010;129(3):427‐436. 19922419 10.1111/j.1365-2567.2009.03197.x PMC2826687

[10] Worbs T , Bode U , Yan S , et al. Oral tolerance originates in the intestinal immune system and relies on antigen carriage by dendritic cells. J Exp Med. 2006;203(3):519‐527. 16533884 10.1084/jem.20052016 PMC2118247

[11] Pabst O , Mowat AM . Oral tolerance to food protein. Mucosal Immunol. 2012;5(3):232‐239. 22318493 10.1038/mi.2012.4 PMC3328017

[12] Mowat AM . To respond or not to respond—a personal perspective of intestinal tolerance. Nat Rev Immunol. 2018;18(6):405‐415. 29491358 10.1038/s41577-018-0002-x

[13] Friedman A , Weiner HL . Induction of anergy or active suppression following oral tolerance is determined by antigen dosage. Proc Natl Acad Sci USA. 1994;91(14):6688‐6692. 8022835 10.1073/pnas.91.14.6688 PMC44268

[14] Siewert C , Lauer U , Cording S , et al. Experience‐driven development: effector/memory‐like alphaE+Foxp3+ regulatory T cells originate from both naive T cells and naturally occurring naive‐like regulatory T cells. J Immunol. 2008;180(1):146‐155. 18097014 10.4049/jimmunol.180.1.146

[15] Buettner M , Pabst R , Bode U . Lymph node stromal cells strongly influence immune response suppression. Eur J Immunol. 2011;41(3):624‐633. 21246540 10.1002/eji.201040681

[16] Garside P , Steel M , Liew FY , Mowat AM . CD4+ but not CD8+ T cells are required for the induction of oral tolerance. Int Immunol. 1995;7(3):501‐504. 7794826 10.1093/intimm/7.3.501

[17] Chen Y , Inobe J , Weiner HL . Induction of oral tolerance to myelin basic protein in CD8‐depleted mice: both CD4+ and CD8+ cells mediate active suppression. J Immunol. 1995;155(2):910‐916. 7541826

[18] FELASA working group on revision of guidelines for health monitoring of rodents and rabbits , Mahler Convenor M , Berard M , Feinstein R , Gallagher A , et al. FELASA recommendations for the health monitoring of mouse, rat, hamster, guinea pig and rabbit colonies in breeding and experimental units. Lab Anim. 2014;48(3):178‐92. 24496575 10.1177/0023677213516312

[19] Ahrendt M , Hammerschmidt SI , Pabst O , Pabst R , Bode U . Stromal cells confer lymph node‐specific properties by shaping a unique microenvironment influencing local immune responses. J Immunol. 2008;181(3):1898‐1907. 18641327 10.4049/jimmunol.181.3.1898

[20] Poulter LW , Seymour GJ , Duke O , Janossy G , Panayi G . Immunohistological analysis of delayed‐type hypersensitivity in man. Cell Immunol. 1982;74(2):358‐369. 6762253 10.1016/0008-8749(82)90036-3

[21] Waksman BH . Cellular hypersensitivity and immunity: conceptual changes in last decade. Cell Immunol. 1979;42(1):155‐169. 312141 10.1016/0008-8749(79)90229-6

[22] Coombes JL , Siddiqui KR , Arancibia‐Cárcamo CV , et al. A functionally specialized population of mucosal CD103+ DCs induces Foxp3+ regulatory T cells via a TGF‐beta and retinoic acid‐dependent mechanism. J Exp Med. 2007;204(8):1757‐1764. 17620361 10.1084/jem.20070590 PMC2118683

[23] Sun CM , Hall JA , Blank RB , et al. Small intestine lamina propria dendritic cells promote de novo generation of Foxp3 T reg cells via retinoic acid. J Exp Med. 2007;204(8):1775‐1785. 17620362 10.1084/jem.20070602 PMC2118682

[24] Katagiri K , Arakawa S , Kurahashi R , Hatano Y . Impaired contact hypersensitivity in diet‐induced obese mice. J Dermatol Sci. 2007;46(2):117‐126. 17350227 10.1016/j.jdermsci.2007.01.008

[25] Hammerschmidt SI , Ahrendt M , Bode U , et al. Stromal mesenteric lymph node cells are essential for the generation of gut‐homing T cells in vivo. J Exp Med. 2008;205(11):2483‐2490. 18852290 10.1084/jem.20080039 PMC2571923

