# Supplementary figures and images for: Diet‐induced obesity results in impaired oral tolerance induction
Source: Immun Inflamm Dis. 2022 Nov 18;10(12):e720. doi: 10.1002/iid3.720 (PMC9673425; doi:10.1002/iid3.720)

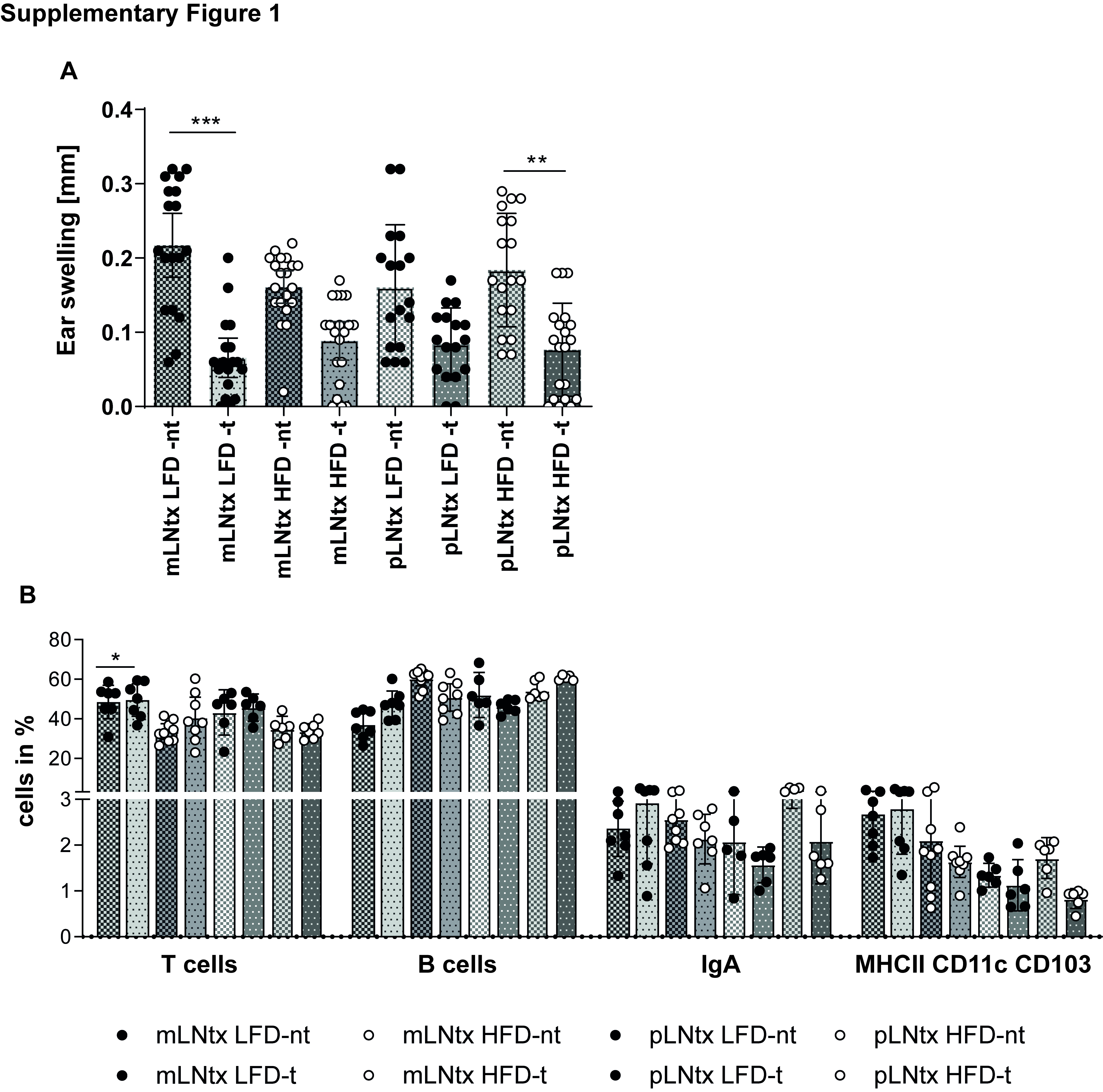

Supplement: Supplementary file 1 — Figurementary Figure 1: Transplantation of mLNs or pLNs was performed, and the mice were allowed to recover for 8 weeks. Afterwards, mice were provided with the LFD or HFD for 10 weeks, and induction of oral tolerance by feeding OVA started. Two independent experiments were performed. A: The DTH response, B: immune cell populations were analyzed (n = 8‐14; mean ± 95% Cl; One‐way‐ANOVA with subsequent Tukey test or Mann‐Whitney‐U‐test). [file IID3-10-e720-s001.tif]
